# Remarks on Blood-letting

**Published:** 1846-12

**Authors:** 


### BUFFALO MEDICAL JOURNAL

AND

### MONTHLY REVIEW.

**VOL. 2.** DECEMBER, 1846. **NO. 7.**

### ORIGINAL COMMUNICATIONS.

ART. I.—*Remarks on Blood-letting.* By the EDITOR.

It is due to the reader, and to ourselves, to state, that the following remarks were written three years ago, and read, by appointment, before the Medical Society of this county. We beg to assure our readers that we do not give it insertion in our Journal from the promptings of self-esteem, or any egotistical estimation of its merits, but solely because we have thought that it may furnish suggestives for reflection upon (as all will admit) one of the most important subjects of Practical Medicine.

The following remarks are submitted in view of the following inquiry:—*What are the general principles determining and regulating the application of blood-letting to the various forms and conditions of disease, which are most in accordance with the present state of medical science?*

It is needless to premise that the limits which I must prescribe to myself will preclude full consideration of a topic like this, which embraces a wide range of investigation, and admits of lengthened discussion. My object will be to present, as briefly as practicable, the general views which have resulted from what examination and reflection I have been able to bestow upon the subject, without professing to adduce all the reasons which appear, to my own mind, to confirm their correctness, and without pursuing the subject in all its various relations and bearings.

It may appear, at first view, that this is a trite topic to bring to the notice of practical physicians. On further consideration, however, it must be apparent that there are few subjects in the domains of practical medicine, which, in the present condition of Pathology and Therapeutics, are more deserving of renewed investigation and discussion. That blood-letting is a remedial agent always potent, and, as regards its energy, often (to use the epithet applied to it by Dr. Clutterbuck) *herculean*, all will admit; and that the practice of different physicians exhibits great discrepancy of opinion respecting its efficacy and appropriateness as a therapeutic remedy, is equally certain. It is probable that here, as in all similar cases, *too much* is attributed to its influence by some, and *too little* by others. It is manifestly, then, a subject, not only open for discussion, but which it is highly necessary to discuss, provided there be any reason to hope that discussion may elicit any new considerations of important bearing, or will enable us to settle our views upon a firmer basis.

It is obviously useful to take into view, at the outset, the physiological, or rather, pathological effects of the loss of blood by blood-letting or otherwise, when it occurs in a healthy state of the economy. Some experiments have been made upon inferior animals to determine these, by Marshall Hall, a brief account of which is contained in Hope's work on diseases of the heart, and the same distinguished pathologist has devoted much attention to the phenomena due to hemorrhage in the human subject. Majendie, also, in his lectures on the blood, presents a variety of facts and observations bearing on this point.

A certain amount of the vital fluid can be withdrawn from the vessels without any notable result. We will not endeavor to estimate the precise quantity required to produce a pathological condition, which, of course, will be affected by numerous varying circumstances. When, however, the loss exceeds what the individual can sustain without injurious consequences, there follow debility, muscular and nervous, and, in general, a languid performance of the vital functions. If the diminution exceed certain limits, and, especially, if it be detracted at repeated periods, the blood itself is found to undergo important changes. Its fibrine and red corpuscles, particularly the latter, diminish in their relative proportions to the mass. From this morbid condition, together with the general diminution in quantity, various pathological phenomena may be induced, some of which are manifested in the solids, and some directly attributable to the perversion of the sanguineous fluid. As belonging to the first we may enumerate nervous irritability, palpitations, or an increased action of the heart producing a peculiar, quick, jerking pulse, called by Hope, the "pulse of unfilled arteries," and constituting what Marshall Hall describes as the "reaction from loss of blood." Cephalalgia, vertigo, tendency to syncope, and other phenomena of the same class, are readily attributable to the deficiency of the universal stimulus, necessary to the healthful exercise of all the vital functions.

Owing to the morbid state of the blood, according to Majendie, and others, may ensue local congestions and extravasations, especially in the pulmonary organs; transudation of the *liquor sanguinis*, constituting the various effusions and profluvia, hemorrhages, &c. The effects of the loss of blood, disconnected from any other pathological condition, are highly interesting, and of great value in their relations to the subject of blood-letting. It is impossible to consider them, on this occasion, as fully as their merits require. The important preliminary consideration to which I would call attention is, that it is practicable to produce artificially, by means of this agent, a great variety of disorders, in themselves of a grave and dangerous character, which disorders not infrequently occur spontaneously in consequence of changes exerted upon the blood by various morbid causes. The elucidation of the pathological phenomena induced by paucity and depravation of the blood has occupied of late much more attention than formerly, and constitutes one of the most interesting features of medical science at the present time. In view of the results of these investigations, we are authorised at this stage of the discussion to say, that modern pathology, if it does not tend to diminish confidence in the efficacy of blood-letting, as a therapeutical agent, most assuredly enforces a necessity of discrimination in its application, vastly greater than seems formerly to have been recognised by writers and practitioners. It is in vain to say of it that it is a remedy which will do no harm, if it does no good. Instances may occur, it is true, in which, within certain limits, it may be safely practiced as an experimental measure, without fear of injury; but such instances are fewer than have been heretofore by many supposed, and with our present knowledge of the pathological phenomena dependent upon the anæmic state, it is by no means difficult to imagine, that when employed under circumstances tending to, or identical with the very morbid condition, which, of itself, it is capable of producing, the injury may be of the most serious character.

It is then a consideration of great consequence, which the practitioner would do well to keep before him whenever he deliberates upon the propriety of blood-letting, that it is an agent possessing power to induce disease in a healthy condition of the organism, and that when injudiciously employed, or inordinately repeated, it may lead to serious and even fatal results. For the facts upon which which this position is based, I refer to the authors already quoted, especially to the lectures of Majendie. This author has doubtless permitted his predilections for the universal agency of the blood in the pathology of diseases to carry him farther than even his own observations will warrant; but, with respect to the general statement just made, his views are confirmed by abundant facts, and sustained by the opinions of the best informed pathologists of all countries.

This consideration, however, is not peculiar to this as distinguished from other therapeutic agents. Almost all remedies are capable of producing injury, or disease, in the healthy subject; but they are employed to meet certain morbid conditions which do not obtain in a state of health, and their virtue consists in obviating these conditions, and thereby placing the patient in a situation more favorable for restorement to health. Now the question arises with reference to blood-letting, are there not certain conditions of disease quite different from the conditions of health, which this agent is calculated to remove, and which, within certain limitations, are, in reality, (if I may use the comparison) antidotes to the pernicious consequences which might follow its employment in the same individuals in a state of health? If the answer to this be affirmative, the inquiry at once follows, in what do these conditions consist? It is manifest that these questions involve the theory or philosophy of *blood-letting;* and if the progress of science have enabled us to arrive at a satisfactory method of reply to them, we shall acquire the possession of certain rational principles which will enable us to understand and appreciate this remedy more fully, and to carry it into its practical therapeutic applications with far more confidence, promptness, and, if necessary, judicious boldness, than we should be authorized by mere empirical rules, deduced solely from our limited individual observation of its apparent efficacy in various diseases. I proceed to the considerations which these inquiries suggest.

An increase of the mass of blood, under the title of *plethora*, has always been recognised by medical observers as a pathological conditlon. Heretofore it has been supposed that in this condition the normal proportion of the several constituents of the blood is not necessarily impaired; but late researches go to show that a disproportionate excess of the blood corpuscles is characteristic of the plethoric state. This conclusion has been drawn from the observations of Andral and Gavarret upon the blood of those pronounced fair subjects of this condition. While the correctness of these observations is not doubted, nor, in the main, the validity of the deductions, it is still highly probable that a superabundance of sanguineous fluid may frequently exist without this relative increase of the blood corpuscles. The discussion of this question, however, is not relevant to my present purpose, as the remarks with reference to the subject will for the most part be applicable in either case.

It is understood then by plethora to denote an unnatural fulness of the vascular system, from an increase of their contents, with, probably in most instances, a disproportionate excess of the red corpuscles. It is the opposite of *Anæmia*, and might be called general *hyperæmia*. *Polyæmia* is, also, used in a similar sense. Every practitioner has frequent occasion to observe this condition, and the circumstances inducing it are generally sufficiently intelligible. The proximate cause is, on the one hand, excessive activity of the functions contributing to the formation of the sanguineous fluid, conjoined perhaps, with too abundant supply of nutriment, or in too highly a concentrated form; or on the other hand, a diminished activity of secretory and excretory functions. The most common remote cause is a sudden change of habits, from those of physical activity to luxurious indolence, or to sedentary occupations. As the relative proportion of blood to the solids differs in different individuals in a state of health, a tendency to this hyperæmic condition, as also its opposite, in some persons is apparently dependent on a constitutional predisposition, or a peculiar diathesis.

An overplus of the sanguineous fluid, considerably beyond the quantity which stands in a normal relation to the vitality of the tissues, and the necessities of the system, constitutes a condition which has always been regarded by pathologists as highly favorable to the development of numerous diseases — such as congestions, effusions of blood, inflammation—affecting especially those parts of the system which receive large supplies, whose functions are most active and constant, and which are delicately organised. The particular symptoms indicating this condition are sufficiently familiar. I will only observe in this connection, that we find under these circumstances what has been called the "oppressed pulse," occasioned by the imperfect reaction of the distended left ventricle upon its contents. We can easily conceive, upon mechanico-vital principles, that this may be a common cause of hypertrophy of the heart, with or without, but especially with dilitation.

The removal of a portion of the mass of blood in this condition, for the time that its influence extends, only reduces the quantity to its normal standard. In so far it is strictly applicable on rational principles. It is not obnoxious, from its direct effects, to the dangers of diminishing the mass of blood in a state of health, in which the quantity bears an exact ratio to the properties and functions of the organism; although, if we were treating of the subject comprehensively, we should have to take into consideration various modifying circumstances, one of the most important of which is, the existence of a fictitious habit, which the system acquires when subjected to the over stimulation of an excess of blood for a considerable period.

Blood-letting is often demanded in this condition to avert threatened evils of a serious nature. We have all had frequent opportunities of observing immediate and palpable relief from its application. The patient experiences a sensation as if an insupportable weight heen removed. The whole body feels lighter and invigorated. The intellect is more clear, respiration more free, the pulse becomes developed, a peculiar præcordial uneasiness disappears, and if the prophylactic importance of the benefit could be appreciated, we should perhaps perceive that an apoplexy, a hemoptysis, or, possibly, an inflammatory affection had been averted.

The abstraction of blood, however, in such cases, it is obvious, is a measure of expediency only; not, in itself, a true remedy. The cure embraces the proper measures to prevent rapid sanguification, by regulating the quantity and quality of the alimentary supplies; or, what is generally more judicious and efficient, the reformation of the physical habits which have induced the sluggish performance of the functions regulating the expenditure of the blood in the processes of assimilation and elimination.

Of the propriety of blood-letting, when, from an increase in the quantity of blood, secondary disorders of a serious import are threatened, there can be no doubt; but the subject becomes more complicated when considered in connection with the two forms of disease in which the question concerning its importance, necessity, or admissibility, daily presents itself to the practicing physician:—viz., *Inflammation* and *Fever*. I presume they who will read these remarks can recal, within their recent experience of these diseases, numerous cases in which it has been a matter of anxious deliberation to determine whether its employment or omission will be most judicious. 'Does the case call for bleeding, or shall the bleeding be repeated? Does the severity of the disease demand it? Will the patient bear it? On the whole will the chances of recovery be most increased by practicing it or abstaining from it?' Such are the inquiries which the practitioner is constantly compelled to ask himself in treating febrile and inflammatory affections.

Different practitioners and writers, in such cases of doubt and uncertainty, advocate different principles of action. One writer, for instance, says, 'when I have occasion to doubt, I generally take out my lancet and bleed the patient.' I have heard a similar rule supported by those for whose judgement I entertain the highest respect. Others, however, pursue an opposite course, and when there is much room for hesitation as to its propriety, deem the risk of injury to the patient, greater by employing it than forbearing its use. This variance shows that different physicians entertain widely different opinions respecting its general applicability, and the dangers to be apprehended from its injudicious application. It is certainly desirable that the principles of its adaptation should be settled on so definite and clear a basis, as to be recognised and adopted by the profession with greater unanimity. I do not, of course, presume to offer any conclusions of my own with the expectation that they will supply this desideratum. Let us, however, inquire whether we cannot furnish ourselves with some precise ideas on the subject, which it will be useful to carry with us into our practice, and which will at least, assist us when perplexed and undecided.

I will speak first of blood-letting in connection with *inflammation*, and in this connection, develop as concisely as practicable, the general principles, which, in my own estimation, should regulate the application of this remedy in all morbid conditions. It will afterwards be easy to apply these principles to fevers and other forms of disease.

In order to come directly at the kernel of the subject, let me inquire at once, what do we propose to effect by bleeding in local inflammations? It will be answered, perhaps, that we expect to subdue it, to cut it short, or in the significant, but not very chaste expression of Bouillaud, to *strangle* it. Now can this be accomplished? Are inflammations susceptible of being jugulated? I believe the enlightened experience of the profession generally, at the present day, will sustain the assertion, that the instances in which this effort can be made successful, are extremely infrequent. I am aware that the authority of some high names is in favor of an opposite conclusion. Bouillaud, for example, certainly not a mean authority, asserts that acute pneumonia, and articular arthritis may almost invariably be strangled by the very remedy under consideration, if pushed with sufficient boldness, or to quote his language, practiced *coup sur coup*—that is to say bleeding repeated upon bleeding, until the object is attained. Granting to him and others due deference, if I mistake not the views of the majority of the most experienced and enlightened practical observes do not accord with this doctrine. Certainly, if this were true, (to quote the reasoning of an English writer, I think Dr. Hope) it could not at this day be so much a matter of doubt and disputation, for British practitioners, (and we might add American) have not been over timid in the use of the lancet for the last half century or more. If the discovery had not been made prior to the observations of the French Pathologist, it would, at least, have been, ere this, satisfactorily confirmed by the observations of others. But so far from this, the converse appears to have been determined by the equally extensive and accurate experience of numerous other practitioners.

The pathological views which have heretofore generally prevailed concerning the essential nature of the inflammatory process, naturally led to the employment of blood-letting as the most rational and effective method of cure; until lately, and even now to a considerable extent, inflammation has been regarded as consisting chiefly in accumulation of blood, and perverted circulation in the vessels of the affected part. It is not to be denied that many of the attendant and resultant phenomena are thus attributable to the capillary and supplying vessels, but I believe I am authorized in the assertion, that agreeably to present pathological views, the prime, essential cause is ulterior to the vessels and circulation, being referrible to a modification to that unknown something, which, in our ignorance, we call the vital endowments, properties, or forces—in other words qualities appertaining to the solid tissues, determining their development, renewal and functions, apparently more intimately connected with tne nerves than any other of the organised elements of the body, while the phenomena appertaining to the blood and blood-vessels, are secondary occurrences—*effects* not *causes*.

The nature of inflammation, in so far as we are qualified by the present state of science to understand it, in addition to the surer evidence of accumulated experience, tends to diminish our confidence in this remedy as a means of an annihilating at a blow, or, as Bouillaud has it, by blow upon blow, the inflammatory process after it is once located and established. But is it never thus annihilated? Notwithstanding the results of experience are such as have just been represented, we cannot doubt, as it seems to me, that there are cases which furnish an affirmative answer to this question. It is difficult to demonstrate this, because there is always room for the objection, that the disease would *not* have progressed, had the remedy been omitted, but with due allowance for this objection, all the cases which go to confirm this opinion, it is not reasonable to dispose of in this way. The question which now suggests itself is of great consequence in the discussion of the subject under consideration, viz., what circumstances characterise those cases which admit of being cut short by this remedy, and in what do these circumstances consist? I am not prepared to bring positive and abundant facts bearing on this question adequate a demonstrative conclusion, nor, within my present limits, can I enter upon a discussion of the various matters involved. I must content myself with submitting the views which appear to my own mind most rational and satisfactory, and these extended with certain modifications, will apply to blood-letting in all the forms of disease in which its applicability is a matter of question.

Local inflammations may occur during general hyperæmia. An excess of the circulating fluid (as we have seen) in the opinion of most pathologists, constitutes a condition in which inflammation will be developed by causes which, were the quantity of blood in a normal proportion to the solids, would have no such effect. In this point of view, general hyperæmia stands indirectly, or it may be directly to local inflammations in the relation of causation. Abstracting blood in such cases, strikes at the most important root of the disease. For precisely the same reason that when this condition is seasonably recognised and blood-letting resorted to, inflammations, or other evils will be oftentimes averted, it follows that the inflammatory process, when actually commenced, may be destroyed if its existence depend on this same condition. Most inflammations that are in reality strangled, are, I imagine, of this description. Here then, if this be true, is deducible an important precept. If we can determine that general hyperæmia existed previous to, or consentaneous with the inflammatory attack, we may resort to blood-letting not only with confidence, as the most appropriate remedy, but with an expectation that the successive stages may be prevented.

But does this antecedent or co-existent hyperæmic condition *limit* the utility or applicability of blood-letting? Let us pursue the subject farther, and consider some facts which have an important bearing on this question. Active inflammation, as we all know, induces certain changes in the circulating fluid, the most notable of which is an increase of the relative proportion of its fibrin. The circulation takes place, also, with greater rapidity. Now these circumstances induce a condition nearly equivalent to general hyperæmia, and that these circumstances are concerned to a considerable extent in augmenting the intensity and continuance of the inflammatory process, is more than probable. The abstraction of blood is calculated to meet this condition, and although there is less reliance to be had upon it as a means of cutting it short at once, the severity and dangers may be mitigated or removed by its judicious application.

We have then another practical deduction, viz.—in inflammatory affections accompanied with that group of symptoms ordinarily expressed by the term *active*,—bleeding is applicable upon rational principles. But to what extent? This, in a practicable point of view, is a very important question. To answer it fully would involve details incompatible with my present plan. I can only say, in general terms, that the principle regulating the extent, is to diminish the increased fibrinous condition of the blood, and to reduce the mass, so that with due allowance for the increased rapidity of its motion, not forgetting the dangers of too great depletion, the circulation of a given quantity in a given time shall approximate toward the state of health. To define the limitations resulting from the application of this general principle, on the one hand, and the evils of pushing the remedy too far, on the other, would occupy too much time. I would observe, however, that these limitations are probably often exceeded. We do not here, as in the former case, resort to bleeding with a view to *quench* the morbid process, but to diminish its intensity, and prevent serious lesions. Much might be said on this point, and I cannot forbear to repeat the remark, that I suspect the abstraction of blood under these circumstances is often carried to an injurious extent, the practitioner restricting too much his attention to the local accumulation in the inflamed part, which accompanies and characterises, but does not *constitute* the morbid perversion, and excluding from his consideration the dangers of diminishing the circulatory mass to an extent incompatible with that integrity or activity of the vital forces, which are certainly quite as necessary for recuperative efforts in disease, as for the mere preservation of the organism in a state of health.

It remains to speak of the forms of inflammation which are not accompanied by general hyperæmia, nor by those circumstances, in some degree identical, which are said to give to it an *active* character. Sub-acute and chronic inflammations frequently occur without these conditions, in fact, rarely with them, and sometimes under opposite circumstances, viz, anæmia. Upon general principles I am unable to perceive any sanction for general blood-letting in these cases. Whenever anæmia is complicated, it cannot be otherwise than injudicious and dangerous, and hence it should never be omitted to investigate carefully this point. To determine the fact of its existence will not generally be difficult, but in cases of doubt, it may be useful in diagnosis to employ a trial bleeding, not as Marshall Hall advises, to ascertain the tolerance of the remedy, but to observe the relative proportion of fibrin, and red corpuscles, to the mass. This procedure, which is recommended by Majendie, might, perhaps be resorted to in many instances with advantage. Even when the patient is not anæmic, but the local affection is unaccompanied by hyperæmia or active symptoms, it is probable that blood-letting exerts no efficacy to shorten the duration, or to mitigate the severity of the disease. The risk incurred, therefore, is without any redeeming chances for benefit, and can hardly fail to prove an unfortunate measure.

It may be said that these are mere theoretical conclusions originating in speculation, not derived from actual observation. I appreciate the force of this criticism; but where, I ask, are the data for arriving at any exact principles based upon properly ascertained numerical facts, which exhibit a comparison of the requisite number of observations, wherein patients affected alike with the various forms of inflammation, with similar constitutions, habits, circumstances, &c., have been treated with, and without the application of blood-letting! The necessary facts for such an analysis, and numerical contrast, have not yet been collected, nor is there reason to hope that the subject will very speedily be settled by reference to this experimental test. In the meantime, what are we to do? Our individual experience is necessarily incomplete and limited, and we find the most opposite doctrines supported by different practical men, each one referring with equal confidence to his own experience. Acknowledging the immense advantage of evidence to be derived from recorded observations, sufficiently exact, comprehensive and extended, in the absence of such evidence I can see no other method than to avail ourselves of such light as reason furnishes, bringing to bear upon the subject established principles of Physiology and Pathology, correcting, enlarging, and confirming our conclusions by an impartial survey of our own expe- rience, and the experience of our fellow practitioners.

The general principles regulating the application of blood-letting which I have presented are not without numerous modifications in their particular applications, some of which are nearly exceptional in their characters. These modifications appertain to differences of tissue inflamed, and a variety of circumstances, some of which, in the present state of science, are quite unintelligible. To some of these I will devote a few passing remarks.

We discover a palpable difference in some of the symptoms presented when, in so far as we can judge, a similar amount of the local inflammatory perversion is located in different tissues. This difference is strongly marked, for example, when the serous and mucus membranes are contrasted. When the former are attacked, we have generally a hard, firm pulse; while in mucus membranes it may be soft and feeble; the morbid process in each being the same in degree. The immediate effects, or tolerance of the remedy is much less in the latter than in the former. Experience, also, shows that blood-letting is less effective in the latter than in the former. These facts will, of course, influence the degree and extent of its application.

The explanation of this difference probably involves several considerations. The great point of distinction, however, so far as blood-letting is concerned, consists in the fact that in serous inflammations the mass of the circulating fluid in circulation is greater, and the circulation is generally more rapid. This is due to the greater nervous excitability, including pain, which tends to diminish the activity of the assimilating and excretory functions, and to increase the action of the heart; to its density of texture, admitting of less congestive accumulation, and consequently a less amount having to be deducted from the mass in circulation; and, finally, to the absence of the glandular follicles existing in mucus membranes, furnishing a secretion which is a means of local relief, and adds to the general expenditure. These considerations involve sufficient reasons why blood-letting is more applicable, and may be carried further with safety and benefit in inflammations of the serous tissue. This, then, is one illustration of the modifications of the general principles stated. Another relates to the organ affected, without regard to the tissue. The heart and pulmonary organs stand in a peculiar relation to the circulation of the blood, the first furnishing the chief mechanical power employed; the second performing a necessary function of hæmatosis; each involving constant and uniform motions. On this account, when either or both these organs are inflamed, blood-letting is serviceable in a peculiar mode. Diminishing the quantity of circulating fluid diminishes the labor which these organs have to perform, and thereby secures an approximation toward that state of repose, which is favorable to the resolution and safe termination of all local inflammations. On the same principle blood-letting is employed in various organic diseases of the heart.

But in this point of view the remedy is not without its limitations, which it is hazardous to transcend. By inducing the anæmic state of irritability, or what Marshall Hall terms reaction from loss of blood, the very evils are aggravated which it was designed to mitigate. The heart is excited to more frequent contractions, and consequently the task which the lungs, as well as itself, has to perform, is increased by the greater rapidity of the circulation, notwithstanding the mass of blood is diminished. Dr. Hope has pointed out the dangers arising from this source in the treatment of organic diseases of the heart, according to the plan of Valsalva and Albertini, by copious and repeated bleedings, in conjunction with an extreme rigidity of diet, and demonstrated, theoretically and practically, the great superiority of pursuing a medium course, avoiding the obstacles on either side.

Inflammations of the brain, also, give occasion for some modifications of the general principles stated. Although we do not suppose that the essential cause of any inflammatory affection is located exclusively in the blood or the circulation, it is obvious that the immediate effects which present the most prominent sensible phenomena, and which perhaps are the most directly condusive to injurious and fatal lesions, are located in the blood-vessels of the part and their contents. The consequence of the local determination, congestion, &c., are of the most serious character when the brain, or its meninges are affected, owing to the great proportion of blood which, normally, this portion of the body receives, its extreme delicacy and softness of texture, its inexpansibility from the bony case which encloses it, and lastly its vital relations, more especially through the medulla-oblongata and true spinal system.

It is to my mind quite problematical, whether the abstraction of blood to much extent beyond conformity with the general principles which have been presented, will diminish the probabilities of severe or fatal injury. It is well ascertained that cephalic congestions, and some forms of hydrocphalus, are actually induced, by causes appertaining to anæmia, in which blood-letting is positively a fatal measure. This fact should occasion hesitancy in using great boldness with the lancet after the general indications have been met.

Finally, the general doctrine I have advocated is affected in its practical application by some circumstances of an inscrutable nature, of which, as the present condition of our science furnishes no explanation, I shall simply allude to. Some inflammations are distinguished by peculiarities in their origin, progress and consequences, which give to them a specific type or character. Such are the cases which we call *asthenic.* The causes producing these peculiarities are often general, affecting a great number of individuals, extending over a large territory, or confined to a restricted space, or occurring at particular seasons. They may also, be confined to a single individual. This department of the subject offers an interesting field of investigation and inquiry, but I do not propose to enter upon it. It is sufficient to remark that our present ignorance of the pathological principles presiding over these deviations from the ordinary course and history of inflammatory diseases, precludes so satisfactory an application of rational methods of treatment as we can bring to bear upon other cases. Past experience, cautious investigation and experimental applications, constitute our chief dependence in the alternating and fluctuating forms, which, at different times, seasons and places, inflammatory affections are liable to assume.

